# Supplementary material for: Patterns of Intron Gain and Loss in Fungi
Source: PLoS Biol. 2004 Nov 30;2(12):e422. doi: 10.1371/journal.pbio.0020422 (PMC532390; doi:10.1371/journal.pbio.0020422)
Supplement: Table S1 — Also available at http://genes.mit.edu/NielsenEtAl/. (4.3 MB ZIP). [file pbio.0020422.st001.zip › NielsenEtAl/html/19.html]

AN4666.1.NCU02466.1.MG03088.1.FG09420.1


```
 CLUSTAL W (1.82) Multiple Sequence Alignments - Introns Inserted


Sequence 1: AN4666.1	317 aa
Sequence 2: FG09420.1	368 aa
Sequence 3: MG03088.1	440 aa
Sequence 4: NCU02466.1	310 aa
Alignment Length: 457 aa
Number Identitical Residues: 77 aa
Alignment Score (without introns) 5313


MG03088.1 	MNTRPAIEGPPTRTTLSASFNSDSTCFVVGLTYGYAVFMSRDCVMRTTSD1LRSGVGIAS
NCU02466.1	--------------------------------------------------~---------
FG09420.1 	MNVRPPIEASSSEAVLSVSFNNDASCFSVGLDSG-------------ICD1FNAGIGLVQ
AN4666.1  	--------------------------------------------------~---------
          	                                                            

MG03088.1 	MRGVSNVIGL~VGGGQPARWAPNK~LILWNVQA~NKVYLEISTLLPIRGVQMSMERTIVV
NCU02466.1	------MVSA0FMSRDHAFYEPLA0LVLWDDKS1--------------------------
FG09420.1 	MMGTTNYLAL~VGGGRSPKFAMNK0AIIWDDMK~GKVALELTTLTAVRGVQLGRERIAVV
AN4666.1  	MLGQSNYLAL~VGGGRQPKFPQNK0LVIWDDAK~QKVVITLEFRTSVLGVRLSKSRIVVA
          	  . :. :.  . .   . :      ::*:     .         .  .   . .     

MG03088.1 	LKNSVRVYKFDKKPDLITSYETADNILGIADLSVSGDMLAFPGRTSGQVQLVNFATDTVR
NCU02466.1	----------------------------LCCLSSD--RIAFPGRAIGHVQLVEVETGNVS
FG09420.1 	LQNSVRVYSFTKHPDLLHIYETADNLAGLCCLSDK--KLAFPGRTAGQIQLVELATGNVS
AN4666.1  	LLNSIHTFVFSSPPKKLAVFETTDNPLGLACLGQK--VLAFPGRSPGQVQLVELETGNVS
          	  .:       . ..     .::..  .:. *. .   :*****: *::***:. *..* 

MG03088.1 	IIPAHSSTLASIRFSPDGRLVATASEK~GTLLRVFSTATGGRVIELRRGLDPAKVFSLRF
NCU02466.1	IITAHTSALRAMALSQDGELLATASEM0GTIIRVYATSNCARLYELRRGIDKAIIFSIGF
FG09420.1 	IIPAHSSALKAIALSPDGELLASASEK~GTLIRVYSTSNCAKLAELRRGIDPATIFSLAF
AN4666.1  	IIPAHSTPLRAMALSPDGEVLATASEA0GTLVRIFATSNCAKMAELRRGVDHAIIFSLAI
          	**.**::.* :: :* **.::*:***  **::*:::*:. .:: *****:* * :**: :

MG03088.1 	NPAGTMLACTSDKGTLHLYDIP--------ASNAGNAGNAGAQTATNT--PGVGFSDSDV
NCU02466.1	SPSGKYLACTSDKSTLHVFDVTRPGGTRPITSNGGTAYAAGEPSVTGNNRPSSPYSVASS
FG09420.1 	SHCGTMLACTSDKSTLHVFDVP--------HPRKPGMNRSQQIGTP--------------
AN4666.1  	SPSNNLLAVTSDKSTLHVFNLP--------HPRN--APYSNQQASS--------------
          	. ... ** ****.***::::.         ..      :     .              

MG03088.1 	IVKPPTTKAGNAGNSESSNTNNSSTRWGILGQLPLMPRYFRDVTSFAQITFASVDDSPMP
NCU02466.1	SGGGGGVMVNSNNGGSDMAADDGQGRWGFLSKLPLMPRIFSDPYSFASAKFEMADEPVSN
FG09420.1 	----------------GADAGDGTGKWGILSKIPLMPRLFSDAYSFSSTHFEAGDEAAIG
AN4666.1  	----------------SDDGVN--KKWGILGKIPLLPRVFSDVYSFASAHFELGEEEPGP
          	                .    :   :**:*.::**:** * *  **:.  *   ::    

MG03088.1 	TTSANSPARNTEDLVGVSRRVGSSRNGQPLSSSKDNDYSMNIKPNKGEIGWPTEDEIIVI
NCU02466.1	GSREP-----------LTRDGGLAIIG---------------GPLKGNLGWISETEMVVI
FG09420.1 	GIPFS-----------ESTVLGTS------------------RPPKGVIGWIGDDSLVVI
AN4666.1  	TYAPP-----------LGTVLG--------------------RPPKGVIGWSNDNTILVV
          	                     *                     * ** :**  :  ::*:

MG03088.1 	GAGHDARWERFQLVQE------QTGERNLVRSGWKRFMTQI-
NCU02466.1	GAGRDPKWEKFAIQEGGQQDGYQGGGRRLVRVGWKRYGGETP
FG09420.1 	GAGHDARWEKFVIVDG------EDGKRHCVREGWKRYLGNT-
AN4666.1  	GSGSDGRWEKFVLRDD------EEGKKHCIREGWKKYLGSGS
          	*:* * :**:* : :       : * :. :* ***::  . .
```
